# Boston Journal of Chemistry

**Published:** 1877-12

**Authors:** 


### EDITORIAL.

### BOSTON JOURNAL OF CHEMISTRY.

This very interesting and instructive journal grows better and better as it increases in age. It has been so good for years, that we could hardly see each year how it could be improved; and yet improvement was made each year. Sometimes by enlargement; and always by the introduction of more interesting and useful matter. It contains the cream of the scientific progress of the day, especially all that in any way pertains to chemical science. This journal ought to be in the hands of every member of the profession. Every number of it will be of more value to any educated dentist than the subscription price for one year. We will state, for the convenience of those who do not know, that it is monthly; containing twenty-five or thirty pages, quarto, and published at $1.00 per year.

This is one of the publications that we can most heartily recommend to our readers.

